# A Combination of a Dopamine Receptor 2 Agonist and a Kappa Opioid Receptor Antagonist Synergistically Reduces Weight in Diet-Induced Obese Rodents

**DOI:** 10.3390/nu16030424

**Published:** 2024-01-31

**Authors:** Beatriz Cicuéndez, Javier Pérez-García, Cintia Folgueira

**Affiliations:** Centro Nacional de Investigaciones Cardiovasculares (CNIC), 28029 Madrid, Spain; beatriz.cicuendez@cnic.es (B.C.); javier.perez@externo.cnic.es (J.P.-G.)

**Keywords:** obesity, pharmacological treatment, weight loss, synergistic treatment

## Abstract

As the global obesity rate increases, so does the urgency to find effective anti-obesity drugs. In the search for therapeutic targets, central nervous system (CNS) mechanisms engaged in the regulation of energy expenditure and food intake, such as the opioid and dopamine systems, are crucial. In this study, we examined the effect on body weight of two drugs: bromocriptine (BC), a D2R receptor agonist, and PF-04455242, a selective κ opioid receptor (KOR) antagonist. Using diet-induced obese (DIO) rats, we aimed to ascertain whether the administration of BC and PF-04455242, independently or in combination, could enhance body weight loss. Furthermore, the present work demonstrates that the peripheral coadministration of BC and PF-04455242 enhances the reduction of weight in DIO rats and leads to a decrease in adiposity in a food-intake-independent manner. These effects were based on heightened energy expenditure, particularly through the activation of brown adipose tissue (BAT) thermogenesis. Overall, our findings indicate that the combination of BC and PF-04455242 effectively induces body weight loss through increased energy expenditure by increasing thermogenic activity and highlight the importance of the combined use of drugs to combat obesity.

## 1. Introduction

Obesity is the most common metabolic disorder worldwide, and its prevalence has reached epidemic levels [1]. Projections indicate that the global population’s obesity prevalence will increase and reach approximately 60% by 2030 [2], which highlights the necessity of developing new preventive and therapeutic anti-obesity drugs.

Over the past few years, the search for new treatments for weight loss has led to the development of several drugs. Some of them, including orlistat [3], phentermine–topiramate, naltrexone–bupropion, liraglutide, and semaglutide [4], have received approval from the FDA. In recent years, GLP-1 agonists, such as liraglutide and semaglutide, have evolved, and dual glucose-dependent insulinotropic polypeptides (GIP/GLP-1), dual GLP-1/glucagon receptor agonists, and triple agonists have recently been developed [5]. All these compounds promote weight loss and improve glucose tolerance in patients with diabetes by stimulating insulin production and promoting satiety [6,7]. The strategy employed by two of the other FDA-approved drugs involves the combined administration of substances affecting the central nervous system (CNS). Specifically, phentermine–topiramate combines a sympathomimetic, which suppresses appetite through the elevation of norepinephrine levels, with a gamma-aminobutyric acid (GABA) receptor agonist [6,8]. Another example is naltrexone–bupropion, which combines an opioid receptor antagonist with a dual norepinephrine and dopamine reuptake inhibitor, thereby promoting weight loss [9]. The combination of drugs in the treatment of obesity has prompted extensive research into both homeostatic and hedonic/reward mechanisms controlled by the CNS.

The dopamine system can modulate food intake through both reward (hedonic) and hypothalamic (homeostatic) pathways [10]. Indeed, the availability of the dopamine D2 receptor (D2R) showed a proportional decrease in obese individuals corresponding to their body mass index (BMI) [11]. Specifically, the significance of D2R agonists, namely cabergoline and bromocriptine (BC), to reduce body weight has been demonstrated [10,12], and they have been approved in the United States as an adjunctive treatment for type 2 diabetes [13]. In particular, rodent studies have proven that central administration of BC reduces body weight and fat accumulation, increasing energy expenditure, and promoting thermogenesis in brown adipose tissue (BAT) in obese animals [10]. It also enhances glucose tolerance and reduces levels of fasting and postprandial plasma glucose in individuals with diabetes [12,14].

Another well-known central system involved in hedonic/reward mechanisms is the opioid system, which regulates appetite and energy balance [15]. The μ, δ, and κ opioid receptors (MOR, DOR, and KOR) are a family of G-coupled protein receptors that are extensively spread across the CNS [16,17]. Recent data indicate that opioid receptors could modulate and control energy balance. For example, naltrexone, mentioned earlier, used with bupropion, produces weight loss [18]. This highlights the beneficial effects of using opioid antagonists in conjunction with other treatments for weight management. Specifically, the role of KOR in the control of energy homeostasis has been proven, as it has been observed that dynorphin (an endogenous ligand of KOR) controls food intake by increasing CNS activity [19]. Additionally, KOR controls the metabolic response to a high-energy diet [20], and hypothalamic KOR modulates the orexigenic effects of ghrelin [21] and melanin-concentrating hormone (MCH) [22]. Furthermore, it has been demonstrated that KOR receptors mediate the action of nicotine by inducing thermogenesis and browning [23]. KOR receptors can also ameliorate obesity caused by estrogens by increasing energy expenditure [24], as was demonstrated using a selective pharmacological blocker of the KOR system, PF-04455242 [25].

Exploring combined therapies that leverage synergistic mechanisms to increase energy expenditure is crucial. It is important to consider the existence of cooperative systems, which are essential for the development of innovative drugs to address obesity [26]. For this reason, we decided to explore the synergistic effect of BC, a D2R agonist, and a selective antagonist of KOR named PF-04455242. Both compounds can reduce body weight in animals, but it is currently unknown whether they have combined action on body weight metabolism or energy intake. In this study, we demonstrated that peripheral coadministration of both compounds is able to increase weight loss in diet-induced obese (DIO) rats independently of food intake. Furthermore, we show that the combined action of BC and PF-04455242 on body weight occurs through an increase in energy expenditure, triggering the thermogenic program in the BAT. Due to the synergistic effects of both drugs, we observed a remarkable enhancement in body weight reduction in DIO rats, suggesting a significant advancement in the development of a promising combination therapy for the treatment of obesity.

## 2. Materials and Methods

### 2.1. Animal Model and Diets

Adult male Sprague Dawley rats (8–10 weeks old, 250–350 g) were employed in the study. The rats were kept in an environment with a 12 h light/12 h dark cycle, maintaining controlled temperature and humidity conditions. Throughout the experimental period, the rats were provided unrestricted access to water and either a standard laboratory chow diet (Scientific Animal Food & Engineering; comprising 16% protein, 60% carbohydrate, and 3% fat) or a high-fat diet (HFD) (Research Diets 12492; containing 60% calories from fat, 5.24 kcal/g; Research Diets, New Brunswick, NJ, USA) for 12 weeks. All protocols and interventions involving animals were subjected to thorough review and approval by the Ethics Committee of the University of Santiago de Compostela (15010/14/007) adhering to European Union regulations governing the utilization of experimental animals.

### 2.2. Treatments and Surgeries

#### 2.2.1. Intracerebroventricular Treatment

Rats were anesthetized through an intraperitoneal injection of ketamine (100 mg per kilogram body weight (BW)) + xylazine (15 mg per kilogram BW). A stereotaxic surgery procedure was employed to implant an intracerebrovascular (ICV) cannula in the lateral ventricle of the hypothalamus (coordinates: 1.3 mm posterior to bregma and 1.9 mm lateral to the midsagittal suture at a depth of 3.5 mm), as previously described [27]. After this procedure, the animals were individually housed for a 4-day acclimatation period prior to the experiment, allowing them to recover from surgery. The administration of ICV vehicle (DMSO 100 mM), bromocriptine mesylate (40 μg per rat; Tocris, St. Louis, MO, USA), or PF-04455242 hydrochloride (1.39 μg per rat; Tocris, St. Louis, MO, USA) was performed using a 22-gauge needle (Hamilton; Reno, NV, USA) through the implanted cannulas.

#### 2.2.2. Intraperitoneal Treatment

Rats received an acute (24 h) or chronic daily (10 days) intraperitoneal administration (IP) of vehicle (DMSO 100 mM), bromocriptine mesylate (0.625, 1.25, 2.5, 5, 10, and 20 mg/kg per rat; Tocris, St. Louis, MO, USA), or PF-04455242 hydrochloride (0.3125, 0.625, 1.25, 2.5, 5, and 10 mg/kg per rat; Tocris, St. Louis, MO, USA).

#### 2.2.3. Weight Measurements

In all experiments, daily measurements were taken for food intake and body weight. The animals were euthanatized, and the BAT and liver were weighed rapidly postmortem. Additionally, the 24 h fecal output was measured, and the tissue weights as well as faecal output values were corrected for the body weight of the animal for subsequent analysis.

#### 2.2.4. Nuclear Magnetic Resonance

We recorded body composition, including fat and lean mass, using nuclear magnetic resonance imaging (Whole Body Composition Analyzer; EchoMRI; Houston, TX, USA) as previously described [23,28].

#### 2.2.5. Temperature Measurements and Thermal Imaging

The recording of body temperature was performed using a rectal probe connected to a digital thermometer (BAT-12 Microprobe-Thermometer; Physitemp, Clifton, NJ, USA). The measurement of interscapular temperature was accomplished utilizing a high-resolution infrared camera (E60bx: Compact Infrared Thermal Imaging Camera; FLIR, Wilsonville, OR, USA). The subsequent analysis of the images was conducted using an FLIR Tools-specific software package (version number 5.13) [29].

#### 2.2.6. Indirect Calorimetry

Animals underwent analysis for energy expenditure (EE), respiratory quotient (RQ), and locomotor activity (LA) using a calorimetric system (LabMaster; TSE Systems; Bad Homburg, Germany) [10,27]. The animals were positioned in a temperature-controlled (24 °C) chamber supplied with air circulation. Following the calibration of the system with reference gases (20.9% O_2_, 0.05% CO_2_, and 79.05% N_2_), metabolic rate measurements were recorded at 30 min intervals. Prior to starting the measurements, the animals were acclimated for 48 h, and data collected during the final 48 h were utilized for calculating all metabolic parameters.

### 2.3. Statistical Analysis

Data are expressed as the mean ± standard error of the mean (SEM). Group differences were assessed for statistical significance using a two-tailed unpaired Student’s t-test or one- or two-way analysis of variance (ANOVA) combined with Tukey’s post hoc test. Statistical significance was established at a *p*-value < 0.05 (GraphPad Prism 8.0). Specific statistical information and experimental sample sizes (n) are provided in the figure legends.

## 3. Results

### 3.1. Central Administration of BC and PF-04455242 Decreases Body Weight in Combination and Individually

**Figure 1:**
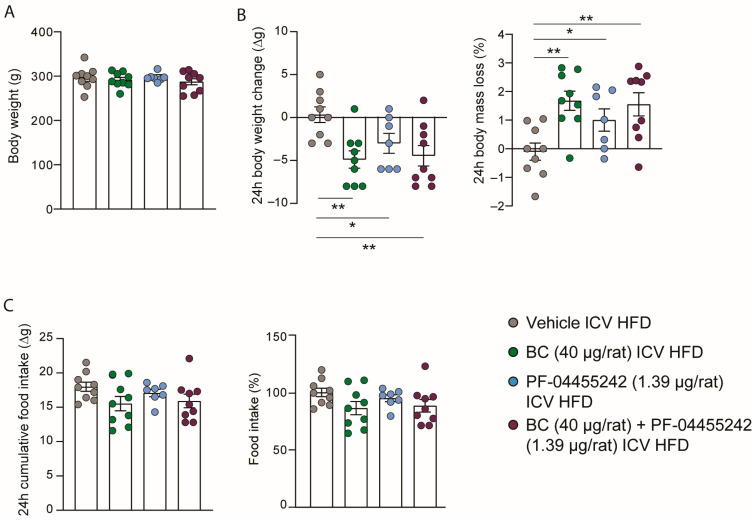
(**A**) Initial weight of the rats evenly distributed among the experimental groups; (**B**) effect at 24 h of intracerebroventricular (ICV) bromocriptine (BC) (40 µg/rat), PF-04455242 (1.39 µg/rat), and BC (40 µg/rat) + PF-04455242 (1.39 µg/rat) injection on body weight change and body mass loss; (**C**) cumulative food intake in 24 h and percentage of food intake compared to control rats in diet-induced obese (DIO) rats. Data are expressed as the mean ± SEM; statistical differences were evaluated using a two-tailed Student’s *t*-test; *n* = 7–9 animals per group. * *p* < 0.05, ** *p* < 0.01 vs. vehicle.

Initially, ICV cannulas were implanted, and the rats were systematically grouped into four categories to ensure uniformity in initial body weight, as depicted in Figure 1A. In keeping with previous studies [10,30], central BC (40 µg/rat) and PF-04455242 (1.39 µg/rat) administration significantly decreased body weight (Figure 1B). To assess whether both compounds could improve body weight in DIO rats when administered together, we ICV coinjected them, and we observed a similar weight loss compared to the single-drug treatments and a concordant increase compared to the control group (Figure 1B). This same pattern is observed when the data are represented as the percentage of body mass loss (Figure 1B). Despite the apparent similarity in the decreasing trend of intake and body weight, the difference in intake is not statistically significant compared to the control group, suggesting that the effects on body weight balance occur in a food-independent manner (Figure 1C). Thus, the effects of the two compounds did not synergize, and we did not find a cumulative reduction in body mass when coadministered centrally in obese animals.

### 3.2. Peripheral Administration of BC and PF-04455242 Increases Body Weight Loss in a Dose-Dependent Manner

**Figure 2:**
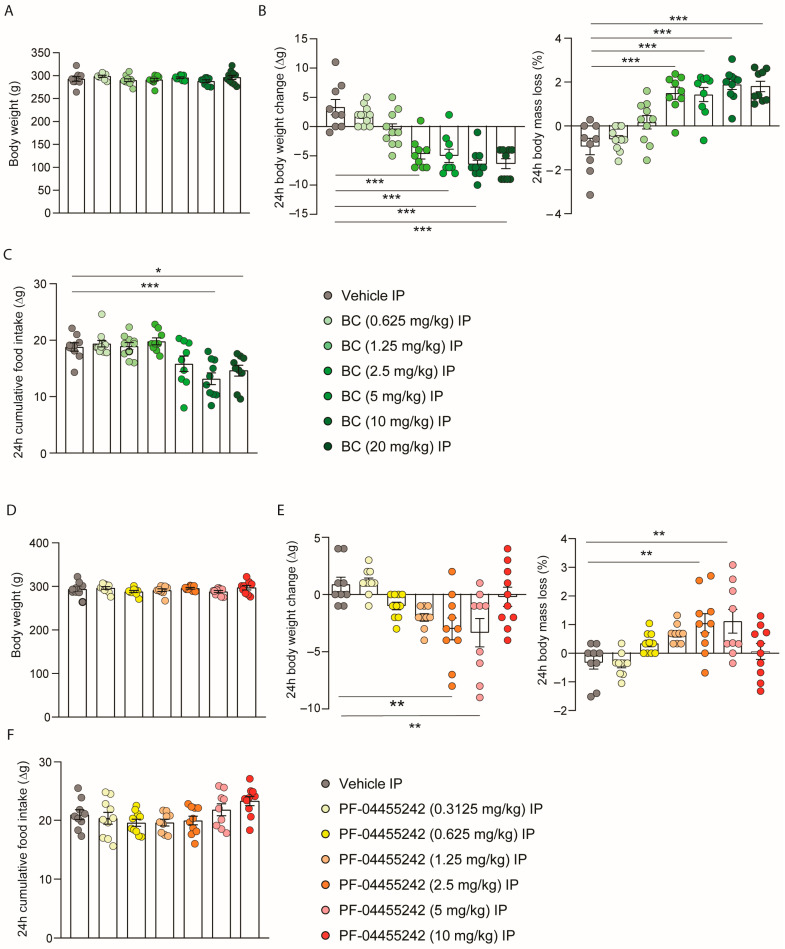
(**A**) Initial weight of the rats distributed among the BC experimental groups. (**B**) Effect at 24 h of a dose-response to intraperitoneal (IP) injection of bromocriptine (BC) (0.625, 1.25, 2.5, 5, 10, and 20 mg/kg) on body weight change and body mass loss and (**C**) cumulative food intake in male rats fed a chow diet (*n* = 9–11 each group). (**D**) Initial weight of the rats distributed among the PF-04455242 experimental groups. (**E**) Effect at 24 h of a dose response to intraperitoneal injection of PF-04455242 (0.3125, 0.625, 1.25, 2.5, 5, and 10 mg/kg) on body weight change and body mass loss and (**F**) cumulative food intake in male rats fed a chow diet (*n* = 9–10 each group). Data are expressed as the mean ± SEM; statistical differences were evaluated using a one-way ANOVA followed by Tukey’s multiple comparisons test; * *p* < 0.05, ** *p* < 0.01, *** *p* < 0.001 vs. vehicle.

Next, we aimed to assess whether these compounds, which were already demonstrated to reduce body weight when administered centrally, were capable of influencing body weight when administered peripherally. Initially, a homogeneous distribution of the animals’ weights was carried out to ensure no initial differences in the peripheral BC dose-response experiment (Figure 2A). A single intraperitoneal (IP) injection of BC (2.5 and 5 mg/kg) significantly reduced body weight and increased body mass loss after 24 h independently of food intake (Figure 2B,C), while higher doses of BC (10 and 20 mg/kg) reduced body weight accompanied by a decrease in food intake (Figure 2B,C). On the other hand, in the dose-response study for PF-04455242, which again was performed with rats with a consistent weight distribution (Figure 2D), we observed that the acute IP administration of PF-04455242 (2.5 and 5 mg/kg) induced a decrease in body weight and, therefore, a higher body mass loss without affecting intake (Figure 2E,F); however, higher doses (10 mg/kg) were incapable of affecting either body weight or intake (Figure 2E,F).

### 3.3. Acute Peripheral Combination of BC and PF-04455242 Causes Synergistic Increased Body Weight Loss in Diet-Induced Obese (DIO) Rats

**Figure 3:**
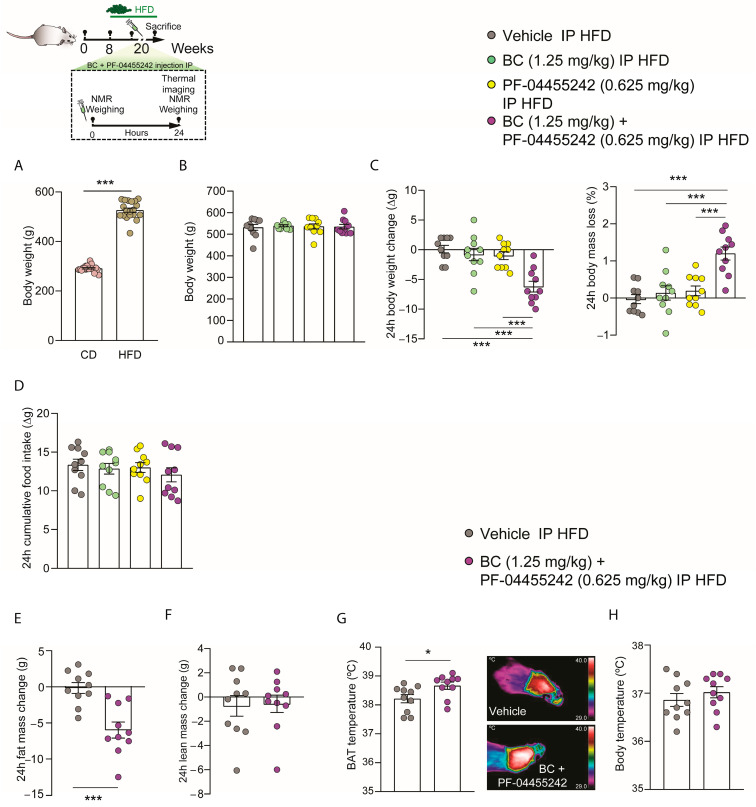
Schematic representation of the in vivo protocol. (**A**) Comparison of total body weight between rats fed a normal diet and those on an HFD for 12 weeks. (**B**) Initial weight of the rats distributed among the experimental groups. (**C**) Effect at 24 h of acute intraperitoneal (IP) bromocriptine (BC) (1.25 mg/kg), PF-04455242 (0.625 mg/kg), and BC (1.25 mg/kg) + PF-04455242 (0.625 mg/kg) injection on body weight change, body mass loss, and (**D**) cumulative food intake in diet-induced obese (DIO) rats. Effect at 24 h of acute intraperitoneal (IP) BC (1.25 mg/kg) + PF-04455242 (0.625 mg/kg) injection on (**E**) fat mass change, (**F**) lean mass change, (**G**) brown adipose tissue (BAT) temperature, and (**H**) body temperature in diet-induced obese (-DIO) rats. Data are expressed as the mean ± SEM; statistical differences were evaluated using a one- or two-tailed Student’s *t*-test; *n* = 10–20 animals per group. * *p* < 0.05, *** *p* < 0.001 vs. vehicle.

According to the previously obtained IP dose-response data for both compounds, we selected the dose of BC (1.25 mg/kg) and PF-04455242 (0.625 mg/kg). We decided to choose these two doses because they represent the minimum doses of BC and PF-04455242 at which we observed a trend toward a decrease in body weight, although without statistically significant changes. Additionally, we aimed to investigate whether, at these doses, the compounds (independently or when administered together) could impact body weight in obesity. To address this, we administered an HFD to rats for 12 weeks, during which we observed a significant increase in their body weight compared to rats on a normal diet (Figure 3A). Next, we organized the rats into four groups with comparable average body weights (Figure 3B). Following the intraperitoneal administration of the two compounds separately to DIO rats over a 24 h period, we observed no significant effect on body weight, body weight loss, or food intake (Figure 3C,D). However, the acute peripheral combination of both BC (1.25 mg/kg) and PF-04455242 (0.625 mg/kg) resulted in a synergistic decrease in body weight accompanied by a consequentially higher body mass loss (Figure 3C) in a food-intake-independent manner (Figure 3D) 24 h after the IP administration of both compounds in DIO rats. These data highlight that the peripheral administration of BC and PF individually at the chosen doses is unable to affect the energy metabolism in both lean and obese rats. By contrast, the combined administration of both drugs at these minimal doses can effectively reduce body weight in obese rats. Therefore, we aimed to investigate whether the cotreatment of rats with BC and PF-04455242 alters body composition. We observed that, in parallel with the increased weight loss, fat mass loss was also significantly higher in IP-cotreated animals for 24 h compared with the control group (Figure 3E), without alterations in lean mass (Figure 3F). Because of the decreased weight gain and adiposity, we next explored the thermogenic profile, and we found a significant increase in the interscapular BAT temperature (Figure 3G), whereas body temperature remained unaltered (Figure 3H). These results suggest that acute BC (1.25 mg/kg) and PF-04455242 (0.625 mg/kg) combination treatment controls body weight by inducing thermogenesis in DIO rats.

### 3.4. Chronic Peripheral Coadministration of BC and PF-04455242 Reduces Body Weight and Adiposity Independently of Food Intake in DIO Rats

**Figure 4:**
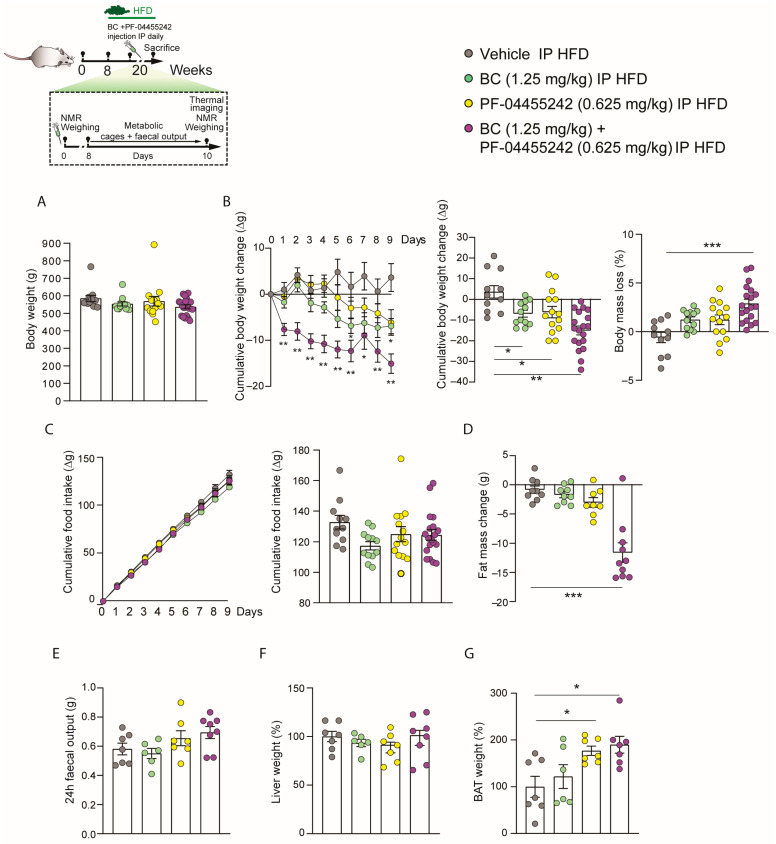
Schematic representation of the in vivo protocol. (**A**) Initial weight of the rats distributed among the experimental groups. (**B**) Effect of a 10-day intraperitoneal (IP) injection of bromocriptine (BC) (1.25 mg/kg), PF-04455242 (0.625 mg/kg), and BC (1.25 mg/kg) + PF-04455242 (0.625 mg/kg) on body weight change and body mass loss (**C**) cumulative food intake, (**D**) fat mass change, (**E**) fecal output, (**F**) liver weight, and (**G**) brown adipose tissue (BAT) weight in diet-induced obese (DIO) rats. Data are expressed as the mean ± SEM; statistical differences were evaluated using a one- or two-way ANOVA followed by Tukey’s multiple comparisons test; *n* = 6–19 animals per group. * *p* < 0.05, ** *p* < 0.01, *** *p* < 0.001 vs. vehicle.

We next investigated whether the effects of the peripheral combination of BC (1.25 mg/kg) and PF-04455242 (0.625 mg/kg) may be long-lasting. Initially, we organized the groups to ensure no differences in weight before initiating the chronic treatment in DIO rats (Figure 4A). Subsequently, we chronically administered both compounds for 10 days in rats fed an HFD. We found that body weight loss was significantly lower in rats treated with BC (1.25 mg/kg) and PF-04455242 (0.625 mg/kg) independently (Figure 4B). By contrast, we observed that the weight loss was much greater when we coadministered both compounds, BC (1.25 mg/kg) and PF-04455242 (0.625 mg/kg), together (Figure 4B). Furthermore, the percentage of body mass loss compared to the control was only significant when we cotreated with the two drugs and not with their individual administration to DIO rats (Figure 4B). However, cumulative food intake did not exhibit any statistically significant differences between treatment groups (Figure 4C). Moreover, we did not observe changes in fat mass with the individual treatment of BC (1.25 mg/kg) and PF-04455242 (0.625 mg/kg) independently, but we found a significant reduction in adiposity after 10 days of peripheral cotreatment with both compounds (Figure 4D) in obese rats. We also evaluated the possible role of the vagus nerve in the body weight changes observed. Interestingly, we did not find alterations in the fecal output in DIO rats (Figure 4E) treated with BC (1.25 mg/kg) and PF-04455242 (0.625 mg/kg), nor did we find changes in liver weight (Figure 4F). Nevertheless, in keeping with the lower body weight seen with the coadministration of BC (1.25 mg/kg) and PF-04455242 (0.625 mg/kg), these animals displayed increased BAT weight in comparison with the control group (Figure 4G). These findings indicate that cotreatment with BC and PF-04455242 increases the activity of BAT in DIO rats, promoting weight loss in a feeding-independent manner.

### 3.5. Chronic Peripheral Cotreatment with BC and PF-04455242 Induces Negative Energy Balance, Triggering Thermogenesis in DIO Rats

**Figure 5:**
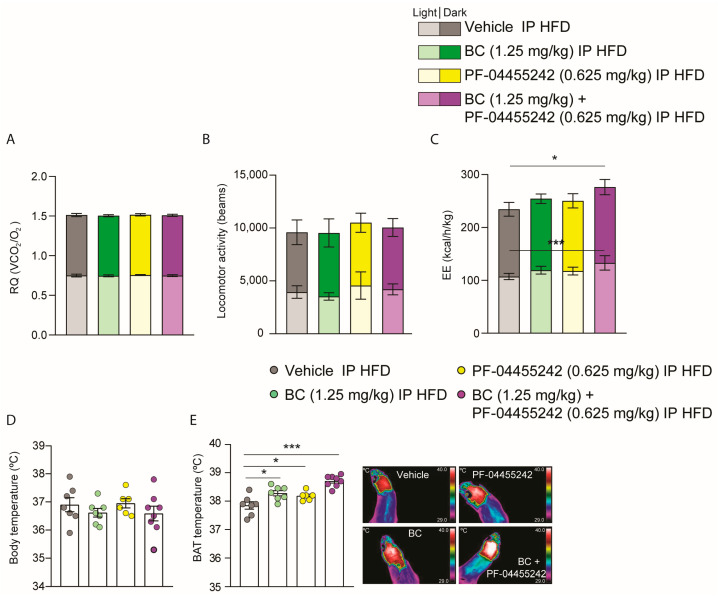
(**A**) Effect of a 10-day intraperitoneal (IP) injection of bromocriptine (BC) (1.25 mg/kg), PF-04455242 (0.625 mg/kg), and BC (1.25 mg/kg) + PF-04455242 (0.625 mg/kg) on respiratory quotient (RQ), (**B**) locomotor activity (LA), (**C**) energy expenditure (EE), (**D**) body temperature, and (**E**) brown adipose tissue (BAT) temperature in diet-induced obese (DIO) rats. Data are expressed as the mean ± SEM; statistical differences were evaluated using one- or two-way ANOVA followed by Tukey’s multiple comparisons test; *n* = 6–8 animals per group. * *p* < 0.05, *** *p* < 0.001 vs. vehicle.

To clarify how the coadministration of BC and PF-04455242 exerted its effects on reducing body weight and adiposity in DIO rats, the animals were monitored through the indirect calorimetry system. No differences were noted in the respiratory quotient (Figure 5A) or locomotor activity (Figure 5B) when compared to animals treated with the vehicle. However, in agreement with the decreased weight, energy expenditure was increased in DIO rats cotreated with BC (1.25 mg/kg) and PF-04455242 (0.625 mg/kg) for 10 days (Figure 5C). Although no differences were found in body temperature (Figure 5D), analyzing the interscapular temperature of BAT reveals that the individual treatment with both drugs, BC and PF-04455242, is capable of increasing BAT temperature (Figure 5E). However, the most remarkable finding is that the increase in BAT temperature after 10 days of treatment was higher in DIO rats treated with BC (1.25 mg/kg) and PF-04455242 (0.625 mg/kg) simultaneously (Figure 5E), resulting in a synergistic achievement of negative energy balance and a heightened activation of thermogenesis in DIO rats.

## 4. Discussion

The present study provides findings that evidence the efficacy of a novel combination therapy involving BC, a D2R agonist, and PF-04455242, a selective KOR antagonist. This synergistic approach was observed to activate BAT thermogenesis, leading to increased energy expenditure. As a result, there was a notable decrease in body weight and adiposity in DIO rats.

While the existing literature lacks direct evidence concerning the effects of these two drugs together, recent studies have indicated that the central activation of their respective receptors independently is involved in the control of energy balance and body weight [10,22,29]. However, when both substances were coadministered centrally in DIO rats, we did not observe a significant decrease in body weight compared to that in animals treated with BC or PF-04455242 independently. Nonetheless, these observations were made only 24 h after central coadministration; continuous administration over a longer period may indeed result in a reduction in body weight.

On the other hand, when we coadministered both compounds peripherally, which is a much less invasive approach, we observed a reduction in body weight both in the short and long term. This change in body weight occurred even though we administered both drugs at a dosage at which no significant changes in body weight were observed when given separately. However, using these compounds together, we found that the DIO rats lost weight, leading to an increased loss of body mass and a reduction in adiposity. These findings suggest a synergistic effect of the combination treatment. It is noteworthy that when administered individually, neither BC nor PF-04455242 induced changes in body weight or adiposity in the rats. Instead, it is crucial for them to be administered together to unlock this synergy, triggering a remarkable reduction in body weight and enhancing weight loss exclusively in the rats treated with the combination. In addition, the observed data on body weight with the chosen doses, which did not significantly decrease body weight in lean rats and did not affect the body weight in DIO rats after 24 h of separate administration, should be highlighted. While it is true that we did not administer both compounds together peripherally to normal-weight rats, given that the focus of this study was to explore new tools and therapeutic targets for weight reduction, once the doses were optimized, we directly administered both compounds, BC and PF-04455242, peripherally together, demonstrating the synergistic effect of both drugs. Interestingly, although some previous studies have suggested that alterations in KOR may modify food intake [15,31], in our research, we did not observe changes in the food consumption with the doses of PF-04455242 used, which were significantly lower than those needed in previous experiments to observe a decrease in food intake.

Kappa opioid receptors exhibit a widespread distribution within the CNS, encompassing regions such as the ventromedial nucleus of the hypothalamus (VMH), the arcuate nucleus (ARC), and other brain regions, including the ventral tegmental area (VTA) [32]. In addition, several studies have identified significant repercussions on energy balance resulting from the central blockade of opioid receptors [20,33]. This includes effects on NPY neurons [34,35], AgRP neurons [36], or MCH neurons [22,30] as well as their interactions with hormones like ghrelin [21]. In most of these brain regions, the dopaminergic system is crucial, suggesting a potential neurochemical overlap between the dopaminergic and opioid systems. Although most studies linking KOR with the dopaminergic system focus on reward signaling or behavior [37,38,39], it has recently been demonstrated that brain regions where D2R is highly expressed play a pivotal role in the central regulation of thermogenesis and body weight control [10,29]. In this study, we observed that the coadministration of a D2R agonist and a KOR antagonist can lead to a decrease in body weight, enhancing thermogenesis in the BAT. However, the neural connections occurring when both compounds are coadministered peripherally remain unknown. Further studies are required to elucidate the specific brain areas and neuronal populations implicated in these effects.

In recent years, there has been a growing concern regarding the discovery of an effective drug for fighting obesity. While several have been proposed, only five are currently approved for use by the FDA: orlistat, phentermine/topiramate, naltrexone/bupropion, liraglutide, and semaglutide. A sixth drug, lorcaserin, obtained approval for weight loss but was subsequently withdrawn from clinical use due to concerns about an elevated risk of cancer [4].

In this search, the importance of coadministering two drugs (as in the case of phentermine/topiramate and naltrexone/bupropion) has become evident, as it seems to be a promising approach to combat this disease. In our study, we observed that the coadministration of BC (approved in the United States since 2009 as an adjunctive treatment for type 2 diabetes [40]) and the KOR antagonist PF-04455242 (which was tested in a double-blind study for the treatment of bipolar disorder in 2010 [25,41]) triggered weight loss in our animals. This could potentially represent a new effective combination therapy against obesity. However, our study was limited to a 10-day treatment period, and further assessment is needed to evaluate the potential side effects of BC and PF-04455242 combination therapy on the rest of the organism.

## 5. Conclusions

This work demonstrates for the first time that coadministration of BC and PF-04455242 exerts beneficial effects in DIO rats, leading to a reduction in body weight and adiposity. These effects stimulate the BAT thermogenic program and increase energy expenditure, indicating that this combination therapy holds promise as a novel strategy for obesity treatment.

## Data Availability

The data presented in this study are available on request from the corresponding author. The data are not publicly available due to privacy reasons.
